# An encrypted network video stream dataset

**DOI:** 10.1016/j.dib.2023.109335

**Published:** 2023-06-22

**Authors:** Jan Fesl, Daniel Sedlák, Tomáš Macák, Marie Feslová, Michal Konopa

**Affiliations:** a Faculty of Information Technology, Department of Computer Systems, Czech Technical University in Prague; b Faculty of Science, Department of Informatics, University of South Bohemia in České Budějovice

**Keywords:** Video stream, Encrypted, Machine learning, Identification

## Abstract

Most of the video content on the Internet today is distributed through online streaming platforms. To ensure user privacy, data transmissions are often encrypted using cryptographic protocols. In previous research, we first experimentally validated the idea that the amount of transmitted data belonging to a particular video stream is not constant over time or that it changes periodically and forms a specific fingerprint. Based on the knowledge of the fingerprint of a specific video stream, this video stream can be subsequently identified. Over several months of intensive work, our team has created a large dataset containing a large number of video streams that were captured by network traffic probes during their playback by end users. The video streams were deliberately chosen to fall thematically into pre-selected categories. We selected two primary platforms for streaming - PeerTube and YouTube The first platform was chosen because of the possibility of modifying any streaming parameters, while the second one was chosen because it is used by many people worldwide. Our dataset can be used to create and train machine learning models or heuristic algorithms, allowing encrypted video stream identification according to their content resp. type category or specifically.

**Specifications Table**

**Table:** 

Subject	Computer Networks and Communications Computer Science Machine Learning

Specific subject area	Encrypted video stream data acquired from network probes within 3 months.
Type of data	Textual (JSON files)
How the data were acquired	The data was acquired in the form of packets from the hardware network probes. The network probes reported the data using the IPFIX protocol to the Apache Kafka-based pipeline. Finally, from the packet records stored in the pipeline, the TCP network streams were assembled, and the JSON output files were generated.
Data format	RAW
Description of data collection	The data was measured approx. 3 months within the real computer network consisting of active network devices, i.e., routers and switches connected to the optic communication lines. The average achieved network throughput was in tens o megabytes per second.
Data source location	Czech Technical University in Prague, Faculty of Information Technology Thákurova 9, Prague Czech Republic GPS: 50.105116930709194, 14.389857845702709
Data accessibility	Repository name: Zenodo Data identification number: 10.5281/zenodo.8039729 Direct URL to data: https://zenodo.org/record/8039729 The data set is freely available for usage without limitation.

## Value of the Data

• The dataset contains sufficient samples to train machine learning models primarily designed to identify encrypted video streams.
• Data analysts or cybersecurity experts can use the dataset.
• The dataset can be a benchmark for comparing the quality of different algorithms or models designed to identify encrypted video streams.
• The use of the dataset lies in the possibility of speeding up the research, as it took 4 months and required 3 people to create it.
• The dataset is free of malicious content and is freely distributable*.*

## 1 Objective

The main reason and motivation for creating this dataset was the possibility of training and validating different machine learning models to identify encrypted video streams and also that a thematically identical or similar freely downloadable dataset does not yet exist.

The authors of [1] created a thematically similar dataset containing 277 Youtube videos, but it is not structured into categories and cannot be publicly downloaded. The authors' dataset [2] contains 180 videos streamed via the Facebook platform. However, this dataset is also not structured and cannot be downloaded. The authors [3] created a dataset containing 3558 videos from the Netflix, YouTube, Amazon Video, and Vimeo platforms, but even this is not downloadable and does not contain general categories. The authors of [4] created a dataset comprising two types of traffic data, VPN (Virtual Private Network) or encrypted traffic data and Non-VPN or unencrypted traffic. The dataset consists of the data streams (.pcap) of 43 videos and it is downloadable by IEEE DataPort Subscription. The authors of [5] created a publicly available dataset containing different types of traffic, including video streaming. They captured traffic from Youtube (HTML5 and Flash versions) and Vimeo services using Chrome and Firefox. In [6], the authors created a data set containing the network streams adapted for mobile network streaming. The dataset is not structured and targeted for encrypted stream identification.

Our dataset is carefully crafted, freely downloadable, and structured into categories. In addition, it contains data from PeerTube [11], for which detailed information about the streaming platform setup is available, and YouTube, which is a black-box streaming service.

## 2 Data Description

**Fig. 1:**
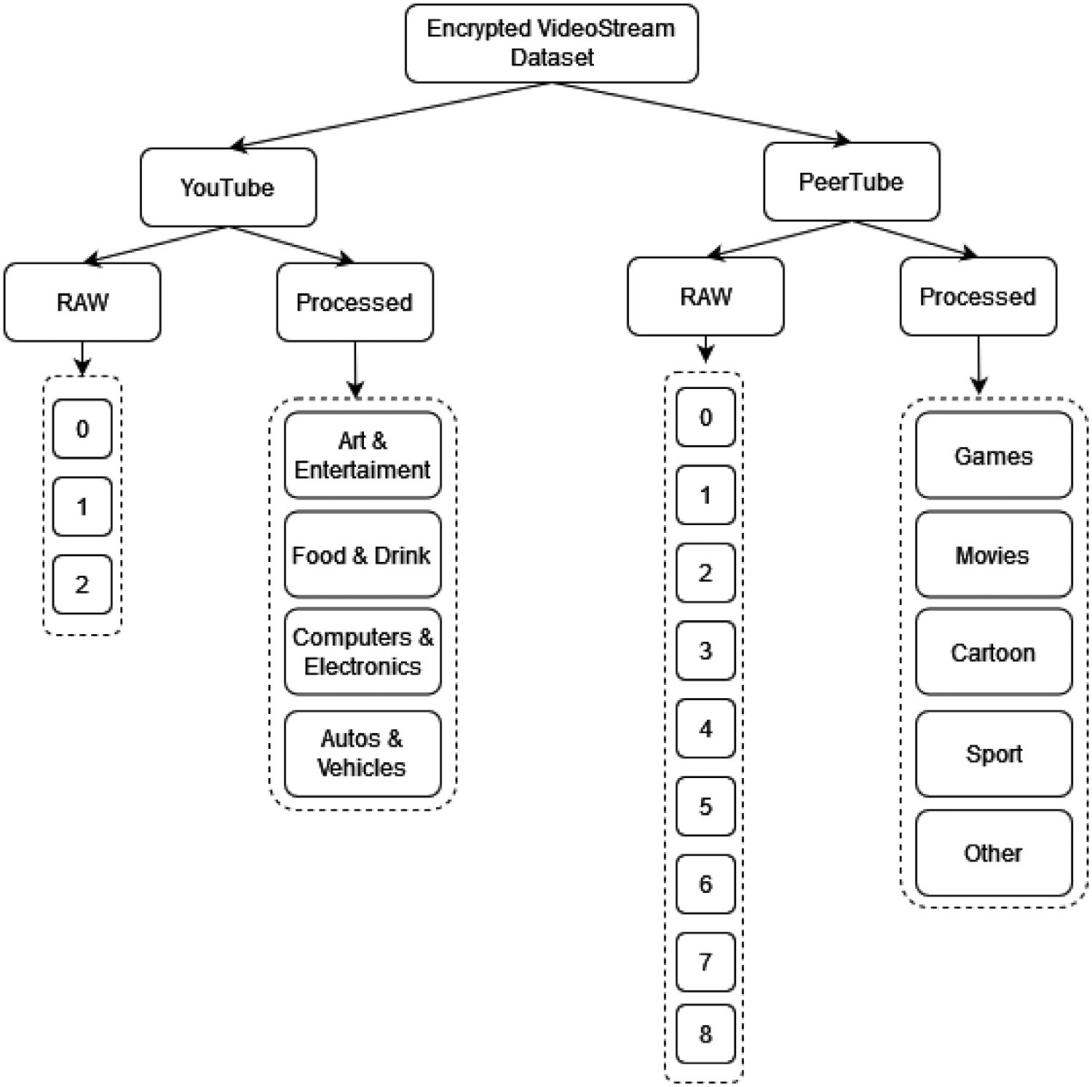
Encrypted video stream data set basic structure. The overall size of the entire uncompressed dataset is cca 96GBs.

The basic structure of our dataset is shown in Fig. 1. As mentioned in the previous section, our dataset contains two complementary parts, namely the measurement of video streams from Youtube and PeerTube platforms. Each part contains the original measured data in the RAW directory in JSON format. The Youtube dataset part contains a total of 2089 examples of video streams measured in 3 repetitions, and the PeerTube dataset part contains a total of 71 examples of video streams measured in 9 repetitions. The PeerTube part deliberately contains a three times higher number of repetitions because the video streams from this part are significantly more extended in time. Thus, the probability of measurement error is higher.

**Fig. 2:**
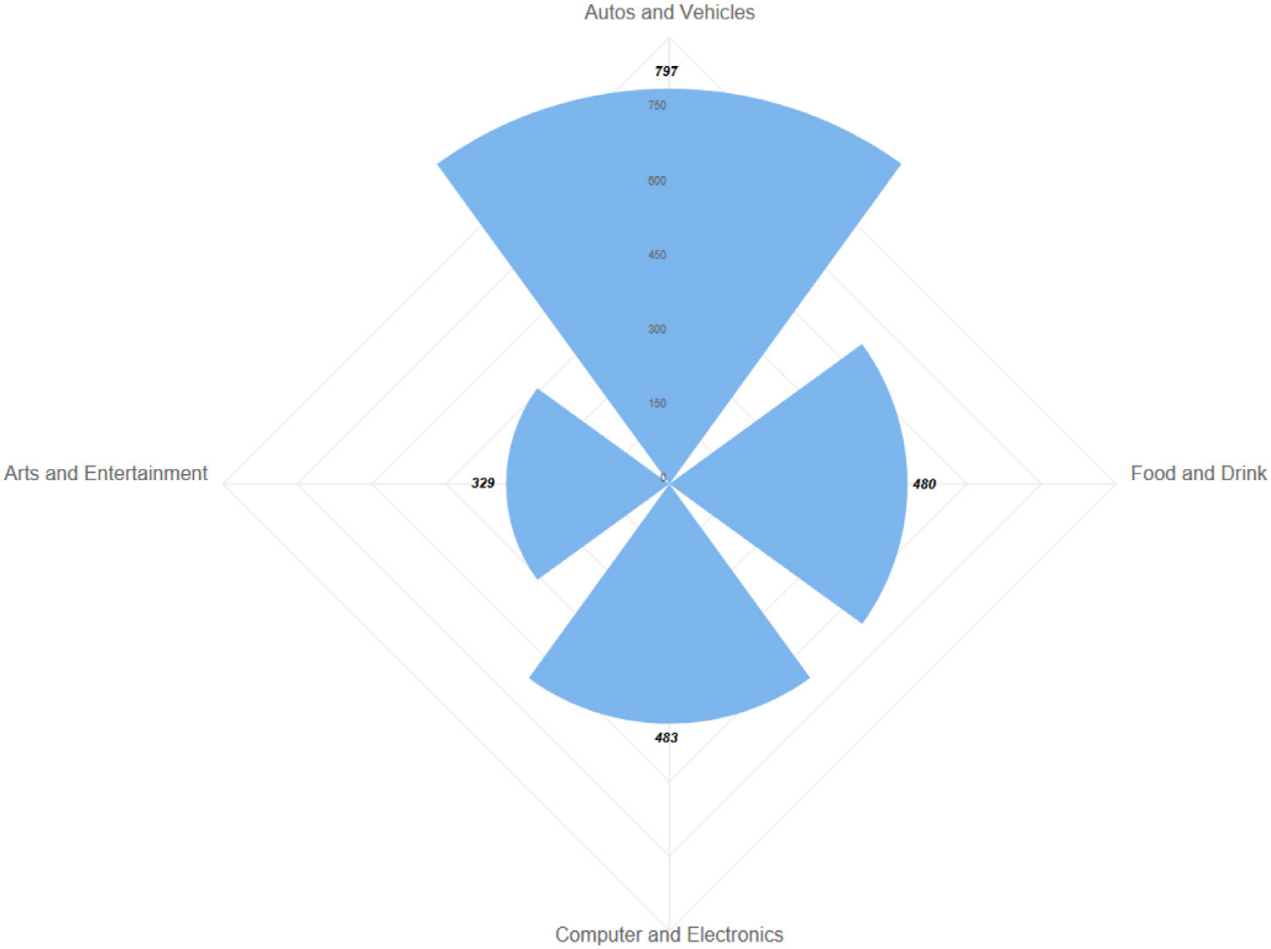
The distribution of measurements for categories in the YouTube dataset part.

**Fig. 3:**
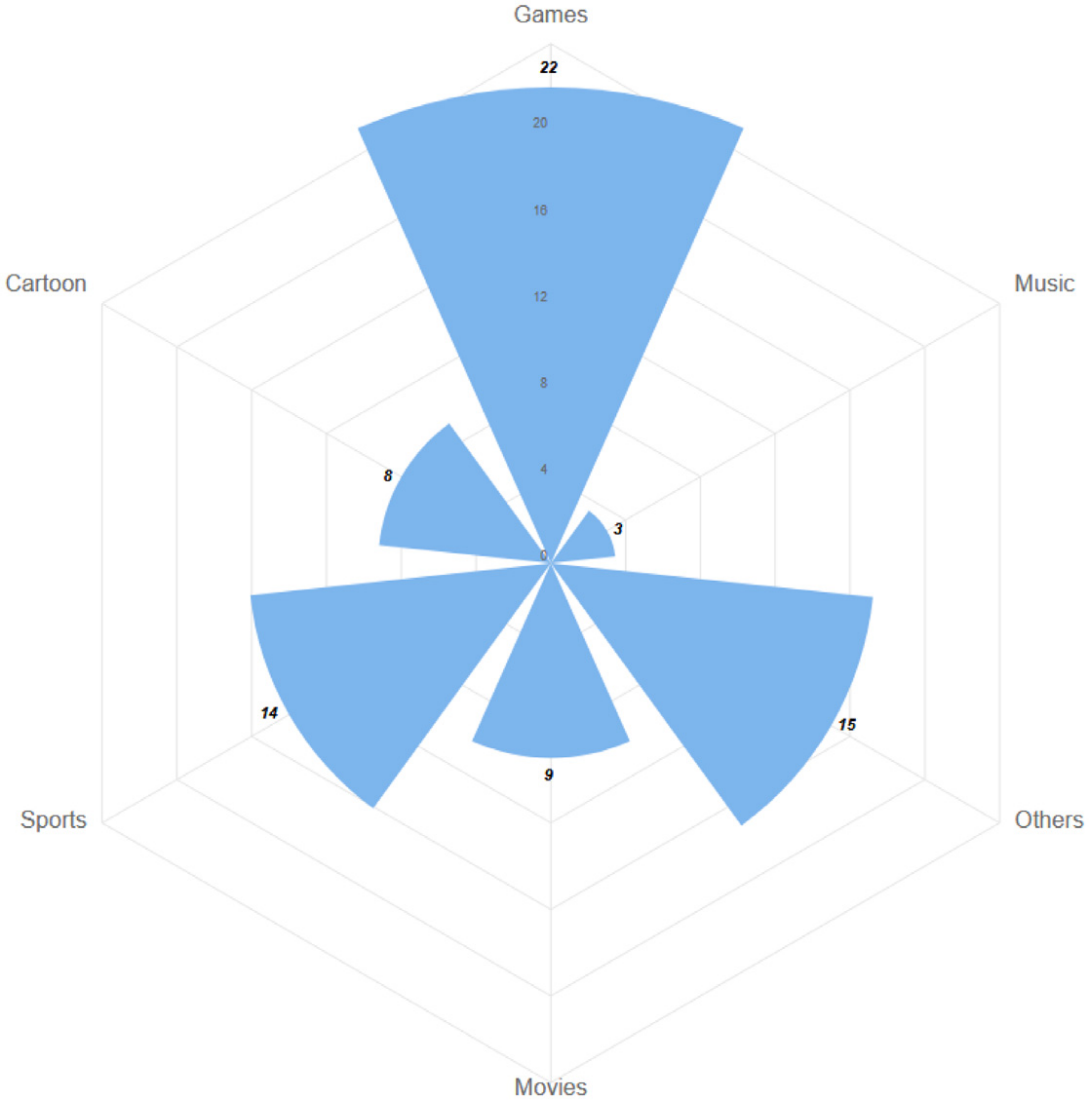
The distribution of measurements for categories in the PeerTube dataset part.

The video streams of the YouTube dataset part is further divided into four sub-parts, namely Art &Entertainment, Food & Drink, Computers & Electronics, and Autos & Vehicles. PeerTube dataset part video streams are divided into only 5 subparts, namely Games, Movies, Cartoon, Sport and Other. The distribution of samples for both dataset parts can be seen in Fig. 2 resp. Fig. 3.

Further, a vector of twenty-five classification features (VCF) has been computed for each video stream. The meaning and explanation of each component of the VCF are presented in Table 1.

**Table 1:** Vector of classification features preprocessed for each video stream present in the data set.

Num	Name	Meaning
1	Request Packets Count	The count of packets sent from the video stream client to the video stream server.
2	Response Packets Count	The count of packets sent from the video stream server to the video stream client.
3	Stream Duration	The total time of video stream duration in milliseconds.
4	Total Request Packets Size	The sum of sizes of all packets sent from the video stream client to the video stream server.
5	Maximal Request Packet Size	The size of the largest packet sent from the video stream client to the video stream server.
6	Minimal Request Packet Size	The size of the smallest packet sent from the video stream client to the video stream server.
7	Median Request Packet Size	The median size of packets sent from the video stream client to the video stream server.
8	Mean Request Packet Size	The mean size of packets sent from the video stream client to the video stream server.
9	Total Response Packets Size	The sum of sizes of all packets sent from the video stream server to the video stream client.
10	Maximal Response Packet Size	The size of the largest packet sent from the video stream server to the video stream client.
11	Minimal Response Packet Size	The size of the smallest packet sent from the video stream server to the video stream client.
12	Median Response Packet Size	The median size of packets sent from the video stream server to the video stream client.
13	Mean Response Packet Size	The mean size of packets sent from the video stream server to the video stream client.
14	Mean Request Packets per Second	The mean count of packets per second sent from the video stream client to the video stream server.
15	Mean Request Bytes per Second	The mean count of bytes per second sent from the video stream client to the video stream server.
16	Maximal Request Inter Arrival Time	The maximal time interval in milliseconds between two packets sent from the video stream client to the video stream server.
17	Minimal Request Inter Arrival Time	The minimal time interval in milliseconds between two packets sent from the video stream client to the video stream server.
18	Mean Request Inter Arrival Time	The mean time interval in milliseconds between two packets sent from the video stream client to the video stream server.
19	Median Request Inter Arrival Time	The median time interval in milliseconds between two packets sent from the video stream client to the video stream server.
20	Mean Response Packets per Second	The mean count of packets per second sent from the video stream server to the video stream client.
21	Mean Response Bytes per Second	The mean count of bytes per second sent from the video stream server to the video stream client.
22	Maximal Response Inter Arrival Time	The maximal time interval in milliseconds between two packets sent from the video stream server to the video stream client.
23	Minimal Response Inter Arrival Time	The minimal time interval in milliseconds between two packets sent from the video stream server to the video stream client.
24	Mean Response Inter Arrival Time	The mean time interval in milliseconds between two packets sent from the video stream server to the video stream client.
25	Median Response Inter Arrival Time	The median time interval in milliseconds between two packets sent from the video stream server to the video stream client.

An entire list of all video streams, including VCFs for all items in the dataset, is available in the file named **all.csv**. This file is available in the root directory of the dataset. The format of each item is as follows (Table 2).

**Table 2:** Record structure for each video stream present in the data set.

Source	Name	Iteration	Category	VCF
YouTube, or PeerTube	The name of video stream	Number of measurement	YouTube or PeerTube subparts	Description specified in Table 1

Complete measurements of all video streams are available in files in the RAW directory. The files are text files, and the structure of their content is in JSON format. In each JSON file, there is an array of flows. A flow is represented as an object where its principal information is stored, such as protocol name and used ports. The description of the flow object is found in Table 3.

**Table 3:** The description of the flow object attributes.

Attribute Name	Description
ip_proto	Name of the protocol
port_dst	Destination port
port_src	Source port
x_packets	Array of the captured packets in the flow

Inside all flow objects, there is an array of the captured packets during that flow. Each packet is represented as a JSON object. The presence of attributes in packets may differ from one flow protocol to the other. The description of the packet object is in Table 4.

**Table 4:** The description of the packet object attributes.

Attribute Name	Description
bytes	The size of the payload of the packet If the value was positive, it means that the packet was in the forward direction. Otherwise, the packet was in the backward direction
timestamp_start	The start timestamp of the captured packet
timestamp_end	The start timestamp of the captured packet
packets	The number of the captured packets during the capturing timestamp
ip_header_len	The length of IP header
tcp_header_len	The length of the TCP header
tcp_ack_number	The TCP acknowledgment number
tcp_flags	The TCP flags
tcp_seq_number	The TCP sequence number

## 3 Experimental Design, Materials, and Methods

This section describes how we gathered the data and used the environment. The first part of this section describes the YouTube dataset, followed by the PeerTube dataset.

### 3.1 YouTube dataset part

**Fig. 4:**
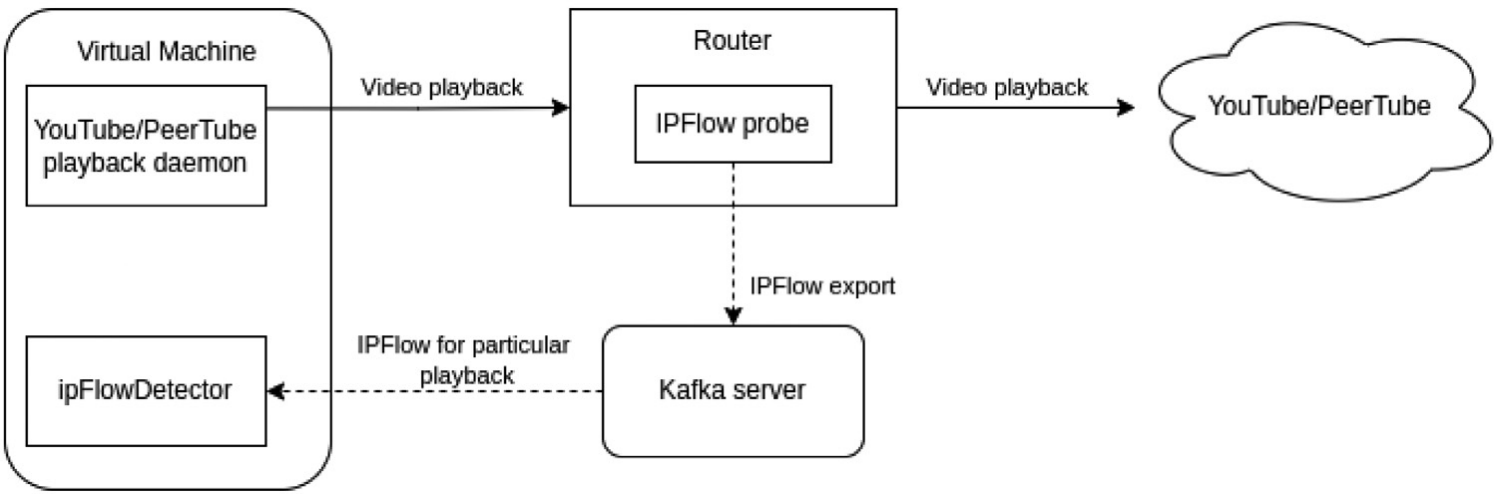
Encrypted streams measurement scheme used for PeerTube and Youtube datasets.

In Fig. 4, we show the scenario used for the video capturing. The figure consists virtual machine, a router, and a Kafka server. The detailed overview of the components and their roles are as follows:

Virtual Machine: The Debian 11.4 server runs the background daemon, which is used for automatic video playback. And the ipFlowDetector, which collects flows for individual videos. The machine has multiple network interfaces to prevent packet interference between YouTube playback and other network traffic.

• Router: A physical router that is dedicated to collecting IPFlows. It hosts a probe that submits collected flows to the Kafka server.
• Kafka server: Kafka [7] stores the captured flows. Probe sends its captured data to a particular topic. The ipFlowDetector then retrieves these data and does further processing.

The YouTube playback daemon in this infrastructure does the primary heavy lifting. It is a service written in the Python programming language, and it accepts a sequence of YouTube videos to play. The videos are played sequentially and from a dedicated interface not used by anything else on the virtual machine to prevent interference. Packets from this interface are routed through a dedicated router that collects the beforementioned IP Flows.

In the main program loop, we iterate over provided YouTube video IDs, and for each ID, we do the following in this order:

• The service resolves from which URL YouTube will start the playback for the given video ID. This is achieved using the yt-dlp [8], which returns the full URL of the video file that will be played. Sometimes we can get multiple URLs.
• Then, we resolve the hostnames of the mentioned URLs of the video file to retrieve the playback server's IP addresses.
• We enforce packet routing for these IP addresses to be routed through the dedicated network interface.
• Next, we spawn the ipFlowDetector, which will collect the flows for the dedicated interface.
• Service spawns a virtual display environment with Full HD resolution, where we will launch the Selenium [9] with the ChromeDriver [10], which starts the video playback.
• We then play the whole video.
• After it is finished, we terminate the ipFlowDetector, which creates a JSON file containing all collected information.

This process is repeated for each video. Furthermore, it is done multiple times for each video.

### 3.2 PeerTube Dataset part

For Peertube video-stream measurement, the same environment described in Fig. is used. The whole process is also done by a program written in Python with the use of ipFlowDetector and Selenium library with ChromeDriver. The main program loop works similarly, as mentioned above in Section 2.1. Still, there are a few differences:

• Before the program is executed, we pass a text file with all URLs of all the videos we want to measure directly to the program as an input argument.
• After the whole page containing the video is loaded, the ipFlowDetector is started with the PeerTube server's IP address as a filter. We can do that because P2P sharing is disabled in the configuration file of our PeerTube server. So, all packets come directly from the server itself.
• All videos uploaded to our PeerTube server are only in Full HD resolution, so we do not need to specify when Selenium plays the video.

## Ethics Statements

Our work does not involve studies with animals and humans.

## CRediT authorship contribution statement

**Jan Fesl:** Conceptualization, Methodology, Writing – original draft, Supervision. **Daniel Sedlák:** Data curation, Software, Writing – original draft. **Tomáš Macák:** Data curation, Software, Writing – original draft. **Marie Feslová:** Methodology, Resources. **Michal Konopa:** Visualization.

## Data Availability

An Encrypted Network Video Stream Dataset (Original data) (Zenodo).
